# Evaluation of Spheroid 3D Culture Methods to Study a Pancreatic Neuroendocrine Neoplasm Cell Line

**DOI:** 10.3389/fendo.2019.00682

**Published:** 2019-10-04

**Authors:** Giulia Bresciani, Leo J. Hofland, Fadime Dogan, Georgios Giamas, Teresa Gagliano, Maria Chiara Zatelli

**Affiliations:** ^1^Section of Endocrinology and Internal Medicine, Department of Medial Sciences, University of Ferrara, Ferrara, Italy; ^2^Department of Internal Medicine, Erasmus Medical Center, Rotterdam, Netherlands; ^3^Department of Biochemistry and Biomedicine, School of Life Science, University of Sussex, Brighton, United Kingdom

**Keywords:** 3D culture, pancreatic neuroendocrine neoplasms, BON1, spheroids, Sunitinib

## Abstract

Pancreatic Neuroendocrine Neoplasms (pNEN) are rare tumors which treatment still represent an important clinical problem, due to the paucity of medical treatments. Due to tumor complexity, techniques as 3D cultures are important to study drug activity in a more realistic model. This study aims to compare three different 3D culture methods in order to understand which one can be considered the best option in terms of experimental easiness and reproducibility in studying the efficacy of a target drug on pNEN. The BON1 cell line was used as a pNEN model and the well-known Receptor Tyrosine Kinase inhibitor Sunitinib was used in order to better investigate the different features of each method. The investigated methods are: (1) 96-well hanging drop plates (HD plates), (2) 24-well plates with a cell-repellent surface, and (3) ultra-low attachment 96-well plates with clear round bottom (ULA plates). The evaluated parameters during the study were: cell seeding, easiness in spheroids formation, morphology, culture maintenance, medium change, spheroids monitoring, picture quality, spheroid perimeter measurement reproducibility error, possibility to perform assays into the seeding plate, overall time of the experiment. Moreover, we investigated how culture methods can influence experimental outcomes evaluating perimeter changes, cell viability and immunohistochemistry of spheroids treated with different Sunitinib concentrations. Results showed that each method has weak and strong points but, considering the easiness of spheroids maintenance and reproducibility results, ULA plates method appears to be the best approach to culture BON1 spheroids and, therefore, to study pNEN.

## Introduction

Pancreatic Neuroendocrine Neoplasms (pNEN) are malignancies arising from the Islets of Langerhans and, therefore, are different from the more frequently occurring exocrine pancreas cancer (1). pNEN represent 7% of all NEN and ~2% of all pancreatic neoplasms with an incidence of 1–2 per 100,000 persons per year (2). pNEN diagnosis is mostly influenced by hormonal hypersecretion that usually leads to early diagnosis due to the manifestation of clinical symptoms (3). However, since up to 85% of patients do not display a specific syndrome, pNEN are often discovered incidentally at advanced stages or due to the presence of local compressive symptoms (2). Surgical resection remains the most effective treatment, but >50% of NEN are not resectable at diagnosis (4, 5). Therefore, alternative approaches are employed, such as biotherapy, targeted therapies, chemotherapy, radiotherapy, Peptide Receptor Radionuclide Therapy (5). It is well-known that Somatostatin Analogs are effective in controlling symptoms and in stabilizing tumor growth in specific settings and the beneficial effects of the approaches available up to now are limited (6, 7). Therefore, other therapeutic options have been actively searched for (5, 8). Indeed, molecular pathways involved in pNEN development and growth have been deeply investigated during the last years, leading to the development of molecular targeted therapies (8). Clinical trials have already demonstrated the efficacy of Sunitinib and Everolimus in pNEN treatment (9, 10). However, side effects or drug resistance may limit the beneficial actions of these drugs (9, 11). In particular, Sunitinib inhibits several receptor tyrosine kinases (RTKs) such as Vascular Endotherlial Growth Factor Receptor (VEGFR), Platelet-Derived Growth Factor Receptor and KIT (12). This drug is frequently employed in the treatment of pNEN, since this tumor type is strictly dependent on VEGFR activation (12, 13). Understanding Sunitinib effects on pNEN has been importantly investigated in the last years also by means of *in vitro* studies, that have been essential in clarifying drug mechanism of action (14). In particular, 3D cell cultures have been employed in the attempt to fill the gap between *in vitro* and *in vivo* systems, due to the possibility of partially recapitulating tumor structure and microenvironment (15). Benefits of 3D cultures are multiple and the most important issue is that this approach provides a more accurate representation of a solid tumor mass (16). As confirmed by Maltman et al. (17), 3D cell aggregation leads to the generation of different proliferation areas and, therefore, to different gene expression patterns and cellular behavior in the spheroid that cannot be replicated in 2D systems. Enhanced cell interaction and crosstalk are additional very important characteristics of 3D cultures that contribute to the generation of a complex microenvironment, that, again, cannot be reproduced in 2D cultures. All these features together may be useful for specific research aspects, that cannot be provided by 2D systems, representing an essential asset for drug discovery research (16, 18). Many 3D culture techniques are available and have been optimized during the last 40 years in order to bridge the gap between monolayers and expansive models (19). Those methods involve different strategies for cell aggregation and, therefore, different tools and methodologies (19). 3D culture methods can be mainly divided into two groups according to the presence/absence of a scaffold. In addition, differences between methods are mostly related to the purpose of the study (20). However, it is unclear which growth culture method is more reliable in the study of drug effects on pNEN. In this study we have analyzed three different “scaffold free” 3D culture methods in order to understand which one could represent the best option in terms of experimental easiness and reproducibility. In order to pursue this aim, we have tested Sunitinib at different concentrations on 3D spheroids from a pNEN cell line, the BON1 cells, obtained with different methods. We choose to employ Sunitinib since this drug has already demonstrated a significant inhibitory effect on BON1 cell viability in monolayer (21, 22). We then evaluated the results of each 3D method according to their different specific features and tried to identify which method is the best to study drug activity in BON1 cells.

## Materials and Methods

### Drugs and Chemicals

Sunitinib was purchased from Selleckchem (TX, USA), dissolved in dimethyl sulfoxide (DMSO) and stored at −80°C as 10 mM stock solution until use.

### Human Cell Line

BON1 cells, derived from human pNEN, were a kind gift from Dr. C. Auernhammer, Medizinische Klinik II, University of Munich, Germany. Cells were grown in 1:1 mixture of F12K and DMEM medium (Euroclone, MI, Italy) supplemented with 10% fetal bovine serum (FBS), 10 μ/ml Penicillin/Streptomycin, referred to as “complete medium” at 37°C in a humidified atmosphere with 5% CO_2_. Experiments were performed within the 7th passage (23).

### 3D Model

3D spheroids were obtained using three different methods.

The first method employs 96-well hanging drop plates (Perfecta 3D, 3D Biomatrix, MI, USA). Cells were seeded at 2.4 × 10^3^ cells/well in 30 μl/well complete medium and allowed to form compact 3D aggregates. Two days after seeding and spheroids formation, aggregates were moved into another 96-well plate and treated with Sunitinib 2.5, 5, and 7 μM. Pictures were taken before adding treatments and before adding MTT solution for assessing cell viability.

In the second method, 500 cells were seeded in a 24-well plate with a repellent surface (CELLSTAR® Cell-Repellent Surface, greiner bio-one, KR, AU) and left on a microplate mixer overnight at 80 rpm. The 4th day after seeding, medium was removed and cells were treated with Sunitinib 1, 2.5, and 5 μM. Treatments were renewed after 3 days in new fresh complete medium. Pictures were taken before adding treatments and then at day 7 and 10 after treatment.

The third method was performed as previously described (24, 25). Briefly, 30 μl complete medium containing 2.4 × 10^3^ cells were seeded in each well in an ultra low attachment 96-well plate (Corning® 96-well Clear Round Bottom Ultra-Low Attachment Microplate, NY, USA). Plates were centrifuged at 300 × g for 3 min and treated with the indicated compounds 72 h later. Treatments were performed adding 70 μl of fresh complete medium with Sunitinib at 2.5, 5, and 7 μM into each well. Pictures were taken before adding treatments and before adding MTT solution for assessing cell viability.

### Spheroid Size Evaluation

Spheroid size was evaluated by measuring spheroid perimeter using Image J software (NIH, Bethesda, Maryland, USA). Results are expressed as mean pixel measure ± S.E.M. vs. vehicle-treated control cells from three independent experiments in two replicates. Pictures for spheroid size evaluation were taken with a Zeiss Axiovert 200/M-based phase-contrast microscope for the second method. EVOS FL Cell imaging System was employed for the first and third method.

### Evaluation of Cell Viability

Variations in cell viability were detected using MTT assay (Sigma Aldrich, St. Louis, USA). MTT assay detects NAD(P)H-dependent cellular oxidoreductase activity, which, according to international literature, mirrors cell viability. This enzyme reduces the tetrazolium dye MTT 3-(4,5-dimethylthiazol-2-yl)-2,5-diphenyltenyltetrazolium bromide to its insoluble formazan. A solubilisation solution (in our case dimethyl sulfoxide) is added to dissolve the insoluble formazan product into a colored solution. The absorbance of the solution is then quantified with a spectrophotometer.

BON1 cells were seeded as spheroids in a 96-well plate as described above and treated 3 days after seeding with the indicated compounds. Control cells were treated with vehicle solution alone (DMSO). Spheroids were incubated with the indicated compounds for 3 days and then 10 μl of 5 mg/ml MTT solution were added to each well. After 1 day, 100 μl of MTT solvent were added to each well and plates were incubated for 4 h in order to solubilize formazan crystals. Absorbance at 570 nm was then measured with GloMax® Explorer Multimode Microplate Reader (Promega Corporation, WI, USA). Results are expressed as mean value ± S.E.M. percent optical density (OD) vs. vehicle-treated control cells from three independent experiments in six replicates.

### Immunohistochemistry

Immunohistochemistry (IHC) was performed as described by Wong et al. (26). Briefly, spheroids were cultured and fixed with 10% formalin, washed in 70% ethanol and embedded in HistoGel (Thermo Scientific, HG-4000-012). After fixation and HistoGel embedding, spheroids were embedded in paraffin and cut in 3 μm layer slides. Subsequently, sections were deparaffinized, dehydrated, and incubated with 1:1,000 caspase 3 primary antibody (Cell Signaling, Danvers, USA) overnight at 4°C. All slides were counterstained with eosin and coverslipped.

### Statistical Analysis

Statistical analyses were carried out using ANOVA after proof of homogeneity of variances and normality tests. Data were analyzed using GraphPad (Prism v-7.0); *P* < 0.05 were considered significant (^*^). For all the other experiments, if not otherwise indicated, Student's paired or unpaired *t*-test was used to evaluate individual differences between the means, and *P* < 0.05 were considered significant.

Experimental reproducibility was evaluated by assessing reproducibility error, represented as the mean difference between the maximum and minimum measurement for each assessed variable.

## Results

### Influence of Sunitinib on BON1 Spheroids Cultured Using a 96-Well Hanging Drop Plate

**Figure 1:**
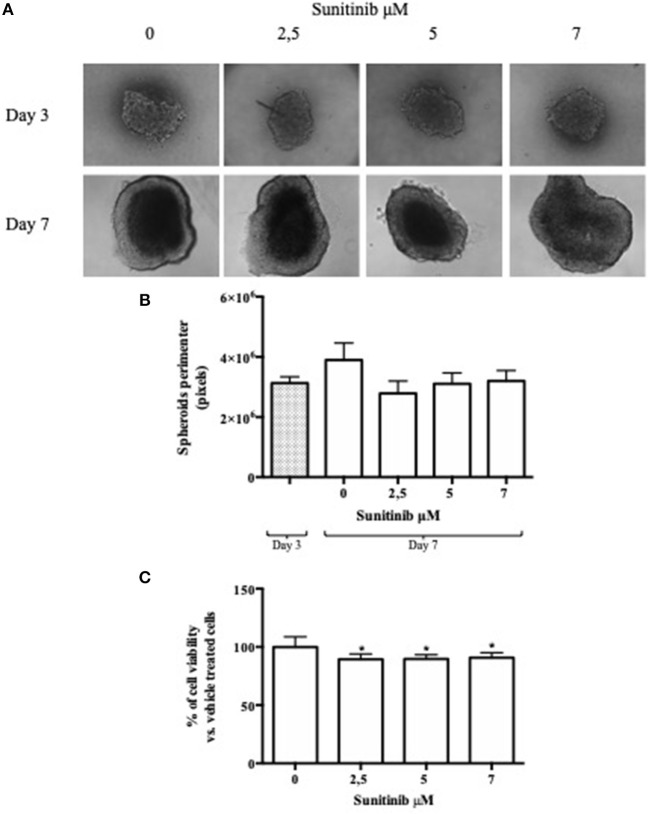
Hanging drop method. BON1 cells were seeded into a 96 well hanging drop plate and treatments with Sunitinib were performed after spheroids transfer in a regular 96 well plate. **(A)** In the upper lane (Day 3) pictures were taken at day 3 after seeding inside the 96 hanging drop plate with EVOS FL Cell imaging System (10 × objective); in the lower lane (Day 7) spheroids pictures were taken at day 7 after transfer in a regular 96 well plate. Spheroids were treated at day 3, after transfer from the first to the second plate, with Sunitinib 2.5, 5, and 7 μM. **(B)** Perimeter analysis of spheroids was performed at day 3 and 7 and represented in a graph. Gray column: perimeter analysis at Day 3, before treatments. White columns: perimeter analysis at Day 7 under indicated treatments. The analysis was performed using Image J software and measurements were performed from three independent experiments in two replicates. **(C)** Cell viability was measured as absorbance in three independent experiments with six replicates each, and it is expressed as the mean ± S.E.M. *P < 0.05 vs. vehicle cells.

BON1 cells were cultured using a 96-well hanging drop plate and treated with Sunitinib at different concentrations chosen on the basis of previous experiments (21). Spheroids pictures were taken before adding Sunitinib (3 days after seeding) and before adding MTT (7 days after seeding). As shown in Figure 1A, pictures taken at day 3, while spheroids were inside the hanging drop plate, are blurred and unclear. This peculiarity is due to the conformation of the plate and to the fact that spheroids are in suspension inside a drop; all these features hamper the possibility to take clear and focused pictures. After spheroids transfer into a 96-well plate with ultra-low attachment bottom (7 days after seeding) pictures appear clearer and more defined. This procedure implies several passages that amplify the likelihood of mistakes, prolongs hands-on time and increase experimental variability (Table 1). Analysis of spheroids perimeter was performed both 3 and 7 days after seeding and is presented in Figure 1B. This evaluation showed that there is no significant difference between mean perimeter of vehicle treated spheroids and mean perimeter of spheroids treated with different Sunitinib concentrations. These results are due to the great variability of the performed measurements, as indicated by a very high reproducibility error (see Table 1). Therefore, this method does not appear to be highly reproducible.

**Table 1:** Comparison of the results obtained for 3D spheroids with the three different methods.

	**3D culture methods**		
	**96-well hanging drop plate**	**24-well plate with a cell-repellent surface**	**Ultra-low attachment 96-well plates**
Cell seeding	Easy	Easy	Easy
Easiness in spheroids formation	Easy	Intermediate	Easy
Morphology	Round type with jagged edges	Round-type	Round-type
Culture maintenance	Difficult	Difficult	Easy
Medium change	Difficult	Difficult	Difficult
Spheroids monitoring	Difficult	Intermediate	Easy
Picture quality	Low	High	High
Spheroid perimeter measurement reproducibility error	2.9 × 10^6^ pixels	N. A.	1.2 × 10^6^ pixels
Possibility to perform assays into the seeding plate	No	No	Yes
Overall time of the experiment	7 days	10 days	7 days

*Reproducibility error is not available for spheroids seeded with the second method since several spheroids were thrown away during medium refreshing*.

At day 7 MTT analysis was performed in order to assess Sunitinib effects on cell viability. As shown in Figure 1C, treatment with Sunitinib significantly reduced cell viability by ~20% at all concentrations tested (*P* < 0.05 vs. vehicle-treated cells).

### Influence of Sunitinib on BON1 Spheroids Cultured Using 24-Well Plates With a Repellent Surface

In order to compare the differences between 3D culture methods, we generated BON1 spheroids with a different method employing a 24-well plate with a repellent surface. Spheroids were seeded and then treated with different Sunitinib concentrations at day 4 and at day 7 after seeding. Pictures were taken before each treatment and before fixation for IHC studies.

**Figure 2:**
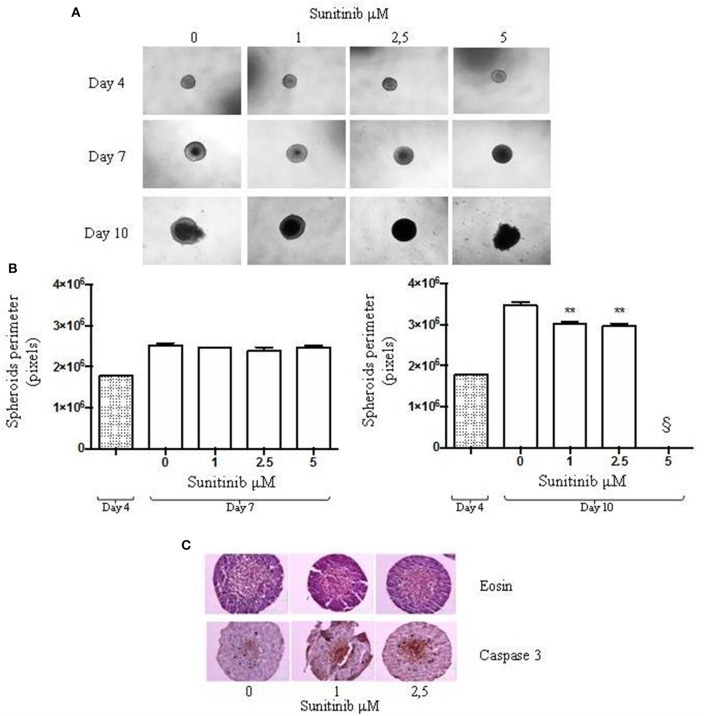
24-well plate with a cell-repellent surface method. BON1 cells were seeded into a 24 well plate with a repellent surface, mixed overnight at 80 rpm. **(A)** Spheroids were treated with increasing Sunitinib concentrations and pictures were taken at day 4, 7, and 10 after seeding with a Zeiss Axiovert 200/M-based phase-contrast microscope (5 × objective). **(B)** Perimeter analysis of spheroids was performed at day 4, 7, and 10. Gray column: perimeter analysis at Day 4, before treatments. White columns: perimeter analysis at Day 7 and 10 under indicated treatments. The analysis was performed using Image J software and measurements were performed evaluating three independent experiments in two replicates. **P < 0.01 vs. vehicle cells at Day 10. § = 5 μM measurement was not detectable for technical reasons, as indicated in the results section. **(C)** Immunohistochemical expression of Caspase 3 in spheroids treated with different Sunitinib concentrations. Spheroids were fixed at day 10 and pictures were taken with a Zeiss Axiovert 200/M-based phase-contrast microscope. Pictures provide an overview of the entire spheroid stained with eosin and Caspase 3 antibody.

In order to understand Sunitinib action on this type of 3D BON1 culture we evaluated changes in spheroids perimeter. As shown in Figure 2A, at day 4 spheroids perimeter was highly homogeneous. No detectable changes in spheroids size were observed at day 7 (Figure 2B, left panel), while at day 10 after treatment with Sunitinib 1 and 2.5 μM spheroid perimeter decreased by ~13 and 15%, respectively (*P* < 0.01 vs. vehicle-treated cells) (Figure 2B, right panel). At day 10 spheroids treated with Sunitinib 5 μM displayed an extremely irregular and loose shape, therefore perimeter analysis could not be performed.

In order to explore further possibilities offered by this culture method, we evaluated Caspase 3 activation by means IHC analysis. As shown in Figure 2C, Caspase 3 IHC can be performed on spheroids treated with Sunitinib 1 and 2.5 μM. IHC of spheroids treated with Sunitinib 5 μM was not performed due to the complete spheroid disaggregation during the procedure.

This method needs medium refreshment (not only supplementation), which means that the medium has to be replaced at least twice before fixation, running the risk of inadvertently discarding spheroids. Reproducibility error could not be calculated due to the loss of a high number of spheroids during the procedure (Table 1).

### Influence of Sunitinib on BON1 Spheroids Cultured Using an Ultra Low Attachment 96-Well Plate

**Figure 3:**
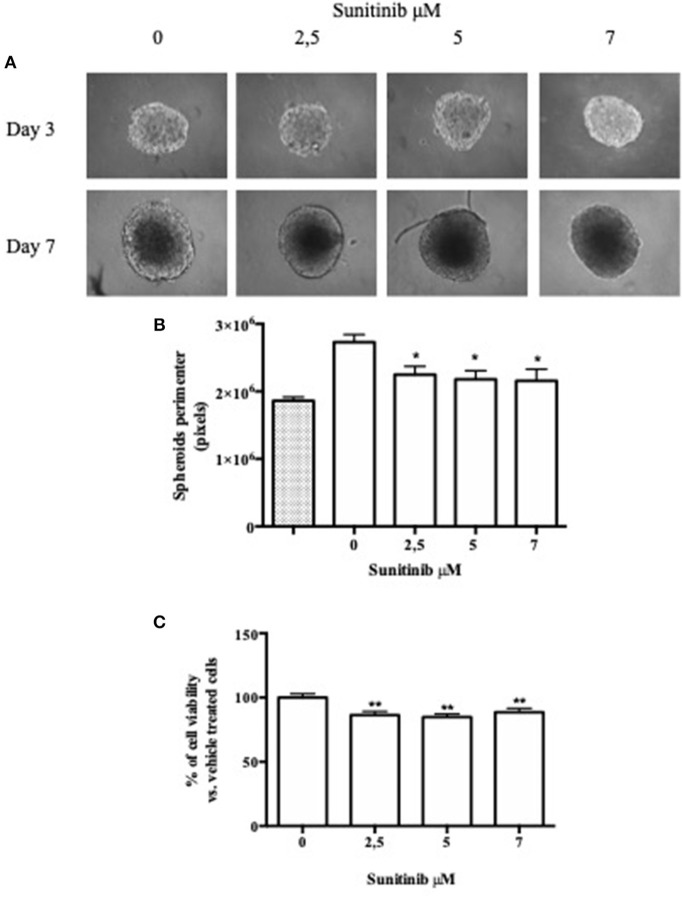
ULA plate method. BON1 cells were seeded into an ultra low attachment 96 well plate and spheroids were obtained by centrifugation. **(A)** Spheroids were treated with increasing Sunitinib concentrations and pictures were taken with EVOS FL Cell imaging System (10 × objective) at Day 3 and 7 after seeding. **(B)** Perimeter analysis of spheroids was performed at Day 3 and 7. Gray column: perimeter analysis at Day 3, before treatments. White columns: perimeter analysis at Day 7 under indicated treatments. The analysis was performed using Image J software and measurements were performed from three independent experiments in two replicates. *P < 0.05 vs. vehicle cells. **(C)** Cell metabolic activity was measured as absorbance in three independent experiments with six replicates each, and it is expressed as the mean ± S.E.M. **P < 0.01 vs. vehicle cells.

BON1 cells were also cultured with a third method involving a 96-well plate with an ultra low attachment bottom (ULA) in order to further investigate the differences between available 3D culture methods. As shown in Figure 3A, spheroids were treated with different Sunitinib concentrations and pictures were taken 3 and 7 days after seeding. The plate conformation improves the quality of the pictures, that appear clear and focused.

Spheroids perimeter was then investigated; we found a significant decrease in this parameter at day 7 after treatment with Sunitinib 2.5 μM by ~18% (*P* < 0.05 vs. vehicle-treated cells) and treatment with Sunitinib 5 and 7 μM by ~21% (*P* < 0.05 vs. vehicle-treated cells) (Figure 3B).

Moreover, as shown in Figure 3C, MTT assay showed that Sunitinib at 2.5, 5, and 7 μM significantly decreases cell viability by ~20% (*P* < 0.01 vs. vehicle-treated cells).

This method does not need spheroid transfer nor medium refreshment, since medium can be supplemented directly in culture wells. Therefore, there is no risk of discarding spheroids. In addition, less replicates are sufficient and reduced incubation times are needed as compared to the previous method in order to observe Sunitinib effects on BON1 spheroids. Variability of the performed measurements is reduced, as indicated by a reproducibility error that is less than half as compared to that recorded for the first method (see Table 1).

## Discussion

**Figure 4:**
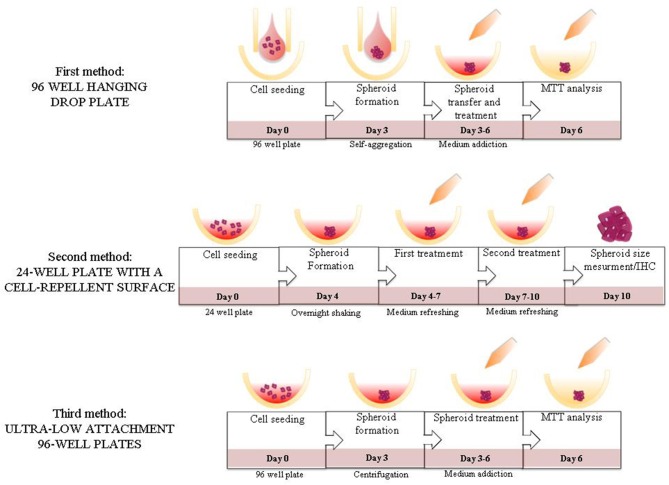
Summary figure. Illustration of the steps of the different methods evaluated in the study.

In the current study for the first time, to the best of our knowledge, three 3D culture methods using a pNEN cell line were compared. We found, as expected, that each method present weak and strong points, as well as different experimental outcomes. Our results indicate that the best option in terms of experimental easiness and reproducibility to test Sunitinib effects on BON1 3D spheroids is represented by the method employing ULA plates (Figure 4).

3D cultures represent an easy and effective way to reproduce a solid mass and mimic tissue key interactions, obtaining more realistic and trustworthy results. Since several methods are available to generate this type of culture, our purpose was to compare different 3D scaffold-free culture systems in order to understand which one could be the best option in the study of pNEN in terms of easiness of culture method and results reproducibility. Therefore, we cultured BON1 cells using three different culture systems.

In the first method, BON1 cells were cultured using a 96-well hanging drop plate. This method is indicated to perform drug screening/cytotoxicity assays and, as underlined by several research groups, its most interesting feature is represented by the exploitation of cell natural tendency to aggregate without scaffolds/matrix involvement (17, 27). The hanging drop method offers several advantages, mainly represented by the easiness of protocol procedures for spheroid formation. On the other hand, according to our experience with the BON1 cell line, spheroids transfer after day 3 was problematic and led to spheroids disaggregation. This observation has been documented also by Amaral et al., who confirm that one of the major weak points of the method is the need to transfer the spheroids to a conventional 96-well plate in order to carry out cytotoxicity assays (28). Structural alterations during spheroids transfer have also been reported by other research groups with different cell lines (29), indicating that this issue appears to be related to the culture method rather then to the cell type. Another disadvantage of this method is the impossibility to refresh spheroids medium due to the small seeding volume (30 μl). Indeed, medium cannot be replaced, since this procedure would imply the presence of a microscope under the cell culture hood to ensure that spheroids are not lost during medium replacement. Therefore, spheroids must be transferred to another plate in order to allow medium/treatment addition. This peculiarity has been documented by several research groups and can represent an important weak point (17, 27, 30). Moreover, it is difficult to find the correct focus while spheroids are located in the hanging drop plate; this feature limits the possibility to take good quality pictures, hampering spheroids perimeter measurements. Indeed, we found a higher reproducibility error for the first 3D method as compared to the third 3D method in terms of perimeter measurement. Since one of the main possibilities offered by this culture method is to perform drug screening and cytotoxic assays we performed MTT assay on BON1 spheroids treated with Sunitinib. Observed cell viability reduction was significant for all the concentrations tested with small variability, indicating MTT analysis as a good method for assessing drug effects on spheroids viability.

The second method involves a 24-well plate with a cell repellent surface. The most important characteristic concerns spheroids size that, being bigger, allows to perform several assays, including IHC. Cell seeding is very easy and involves only plates with a low attachment bottom and a shaker. This method allows to form spheroids with a regular round shape and, due to the flat bottom well, to take good quality pictures. Heterogeneous spheroids proliferation rate, oxygen and nutrients gradients and good cell-cell contacts are important features of scaffold free systems involving the use of a shaker, as also underlined by other research groups (31). However, medium refreshing represents one of the most tricky and challenging aspects of this culture method: spheroids are difficult to locate in the well and, therefore, could be easily discarded during the procedure. Therefore, many replicates are needed to reduce variability of the performed measurements and allow reproducibility. Similarly to the previous method, spheroids were treated with different Sunitinib concentrations in order to measure perimeter variations. This method allows to detect significant differences in perimeter measurements 10 days after seeding, but may not be ideal to assess the efficacy of cytotoxic drugs at high concentrations. Indeed, in these condition spheroids integrity was compromised in our hands, preventing spheroid perimeter evaluation. In addition, Herrera Martìnez et al. recently demonstrated that in BON1 spheroid obtained by using the 24-well plate with cell repellent surface spheroids size does not correlate with DNA content (32). Therefore, spheroids perimeter analysis is not reliable as a measurement of drug cytotoxicity for 3D spheroid cultured with this method. In addition, MTT evaluation is hardly feasible since spheroids should be transferred to a different smaller plate. However, the most interesting feature of this method is the possibility to perform IHC analysis, that allows to explore protein expression/activation directly in the different spheroid areas, helping to better understand drug mechanisms of action.

The third and last scaffold-free method involves an ULA 96-well plate. In this setting, spheroids formation is enhanced by centrifugation that promotes cells proximity. From the very beginning BON1 spheroids appear very compact and with round type morphology; these characteristic have been indicated to be strongly related to a robust cell-cell adhesion, a key feature for 3D models (28, 33). The well shape promotes the formation of a single spheroid for each well, centrally located; moreover, pictures can be easily taken. Similarly to the hanging drop method, spheroids generated with ULA plates are suitable for drug testing with the difference that there is no need to transfer 3D cultures from one plate to another to perform cytotoxicity assays. Spheroids are seeded inside the plate in a 30 μl volume and treatment can be performed directly adding medium into the wells, without transferring the spheroids. This is one of the most interesting features of this method, since the spheroids are treated in the seeding well and, therefore, are not stressed by transfer, avoiding the risk to damage the 3D structure. Spheroids perimeter was evaluated as for the previous methods and its analysis showed significant results. Indeed, a smaller reproducibility error for perimeter measurement in spheroids seeded with ULA plates was found as compared to the hanging drop method, indicating a better reproducibility. Moreover, ULA plates method allows MTT analysis, that appears to show a higher sensitivity in detecting viability variations as compare to the hanging drop method, probably due to the spheroids higher homogeneity.

In conclusion, we found that ULA plates method allows to obtain the most reproducible results when assessing perimeter evaluation in BON1 spheroids as compared to the other investigated methods. In addition, the possibility to generate a single spheroid in each well, that is not disturbed nor altered due to plate transfer/medium refreshment, allows to obtain better results also in following studies, such as viability/cytotoxicity assays.

## Data Availability Statement

The datasets generated for this study are available on request to the corresponding author.

## Author Contributions

TG, GB, MZ, and LH: conceptualization. GB and FD: methodology, software, and formal analysis. LH and GB: validation. GB: investigation and writing original draft preparation. LH and GG: resources and data curation. TG: writing review and editing. GB and MZ: visualization. MZ, TG, and GG: supervision. All authors reviewed the manuscript.

### Conflict of Interest

The authors declare that the research was conducted in the absence of any commercial or financial relationships that could be construed as a potential conflict of interest.
